# Family meal participation is associated with dietary intake among 12-month-olds in Southern Norway

**DOI:** 10.1186/s12887-021-02591-6

**Published:** 2021-03-15

**Authors:** Elisabet R. Hillesund, Linda R. Sagedal, Elling Bere, Nina C. Øverby

**Affiliations:** 1 Department of Nutrition and Public Health, University of Agder, Serviceboks 422, 4604 Kristiansand, Norway; 2 Department of Obstetrics and Gynecology, Sørlandet Hospital HF, Serviceboks 416, 4604 Kristiansand, Norway; 3 Department of Research, Sørlandet Hospital HF, Serviceboks 416, 4604 Kristiansand, Norway; 4 Department of Sport Science and Physical Education, Serviceboks 422, 4604 Kristiansand, Norway; 5 Department of Health and Inequalities & Centre for Evaluation of Public Health Measures, Norwegian Institute of Public Health, Oslo, Norway

**Keywords:** Infant, Toddler, Diet, Diet quality, Family meals, Sweetened beverages, Vegetables, Drinking water, Commercial baby cereal

## Abstract

**Background:**

Family meal participation is associated with healthier eating among children and adolescents. Less is known about family meal participation among infants and toddlers. The objective of the present study was to explore whether family meal participation at 12 months of age is associated with dietary intake and whether a potential relationship differs according to maternal education or child sex.

**Methods:**

Follow-up data from children born to mothers participating in the Norwegian Fit for Delivery (NFFD) trial during pregnancy were used to assess the frequency of intake of 11 dietary items according to frequency of participating in the respective family meals. Dietary differences according to seldom (0–3 times/week) or often (4–7 times/week) participating in each respective meal category were assessed in linear regression models. Potential dose-response associations with frequency of participation in all family meal categories combined were also estimated. Models were adjusted for maternal randomization status, education, and child sex.

**Results:**

The sample comprised 408 children. A total of 74, 53 and 74% had breakfast, lunch, and dinner with family ≥4 times/week, respectively, while 39% had supper and 27% between-meal snacks with family ≥4 times/week. Having family dinner ≥4 times/week was associated with more frequent intake of vegetables, homemade infant cereal, milk, and water, and less frequent intake of commercial infant foods while the other family meal categories were associated with fewer dietary outcomes. For each additional meal category eaten with family ≥4 times/week, frequency of vegetable intake (β = 0.45), water (β = 0.17), and milk (β = 0.09) per day increased, while commercial infant cereal was eaten less frequently (β = − 0.18). The inverse association between family meals and commercial infant cereal was only evident in children born to mothers in the intervention group. Several associations with diet were stronger and only significant among boys.

**Conclusions:**

Being fed in the context of family meals at 12 months of age was associated with a more favorable diet. Including the infant in family meals has potential in the promotion of early nutritional health.

## Introduction

Optimal nutrition is fundamental to healthy growth and development in the first 1000 days of life due to rapid neural and musculoskeletal growth and maturation [1, 2]. The type and variety of food and beverages provided during infancy and toddlerhood may therefore influence life-long health both in terms of biological development and by shaping long-lasting eating patterns [3, 4]. Aspects of diet quality have been associated with dietary, as well as adiposity, cardiovascular and cognitive outcomes at 7–8 years of age [5].

Food preferences and eating patterns are shaped by early feeding experiences, highly influenced by parental feeding practices [3]. Family meals provide a setting of opportunity in this respect, by way of the combination of foods served, the modeling provided by family members, the possibility for communication, and the family rules and rituals adopted from meal interactions [6, 7]. Regular participation in family meals has been associated with multiple health benefits in children and adolescents, including healthier diet and eating patterns and lower odds of eating disorders, overweight and obesity [8, 9]. An updated meta-analysis by Dallacker et al. [10] confirmed these findings and found that the associations were robust to potential influence of country of residence, child age, number of family members participating in the meal, and type of meal. As age was only crudely accounted for in these models (< 11 vs. ≥ 11 years), associations between family meal participation and nutritional health in the youngest sub-groups cannot be inferred.

Few studies have investigated early participation in family meals in relation to nutritional health among infants and toddlers. In a literature review by Verhage et al. [11] confined to children below 3 years of age, structured mealtimes and higher frequency of family meals were associated with more food enjoyment and less fussy and emotional eating. The review only identified two studies reporting associations between frequency of family meals (mostly family dinner) and toddlers’ nutritional health. Fitzpatrick et al. [12] found positive associations between frequency of family dinners and fruit and vegetable intake in a group of children with mean age 2.8 years. Swanson et al. [13] recruited mother-child dyads from a socially disadvantaged area of residence and found that two-year-old children with a more balanced food intake and a more nutrient-dense diet, were more likely to have meals eaten at the table with family. To our knowledge, no studies have been carried out at child age 12 months, an age at which the toddler should be able to eat the same foods as the rest of the family.

Due to social disparities in dietary intake and diet quality [14], nutritional benefits of family meal participation may differ according to parental education and family socioeconomic position. In a meta-analysis of studies addressing family meals and nutritional health in children, Dallacker et al. [10] found that socioeconomic position slightly moderated the association between frequency of family meals and BMI, but not associations with healthy diet, unhealthy diet, and overall diet quality. To our knowledge, potential moderation of associations between family meal participation and dietary intake have not been explored among the youngest children.

An underexplored area in family meal research is whether both sexes benefit to the same extent from family meal participation. Sex-differences in nutritional vulnerability have been documented from conception throughout early childhood and greater awareness of sex differences in nutritional research has been called for [15].

Dallacker et al. [10] included separate estimates for boys and girls in addition to the pooled effect size if findings for boys and girls were presented separately in included studies, but they did not investigate moderation based on sex.

The aim of the present study was three-fold: (i) to investigate cross-sectional associations of family meal participation at 12 months of age with dietary intake in a dataset of first-born children of mothers having participated in a randomized controlled lifestyle intervention during pregnancy, (ii) to identify potential differences in this relationship according to maternal education, and (iii) to explore whether a potential relationship between early family meal participation and child dietary intake differ according to child sex.

## Methods

We used follow-up data from the Norwegian Fit for Delivery (NFFD) trial to investigate the cross-sectional relationship between frequency of family meals and child diet at 12 months of age. NFFD was a randomized controlled trial investigating the effect of a lifestyle intervention during pregnancy on maternal and newborn health [16, 17]. In total, 606 pregnant women were included between 2009 and 2013 and randomized either to the intervention group receiving dietary advice in addition to access to twice-weekly exercise sessions, or to the control group with continued standard antenatal care. After exclusions due to predefined criteria (miscarriage, twin gestations, low pre-pregnancy BMI, and planned relocation outside study area), a total of 591 women participated in the trial at least until birth [17]. The dietary intervention was based on 10 recommendations designed to increase awareness of food choices, with specific advice on portion sizes, regular meal patterns, limiting the consumption of snack foods, and increasing the intake of water, fruits, and vegetables [17]. Dietary counseling was performed by two telephone conversations, accompanied by a brochure detailing the 10 recommendations. The mothers completed questionnaires at inclusion (around week 15 of pregnancy), in late pregnancy (week 36), and six, 12, 24 and 48 months postpartum for themselves and their child. Data on food intake, meal frequency, frequency of family meals and other aspects of dietary behavior were collected at all time points postpartum. The NFFD trial was performed in accordance with the Declaration of Helsinki. The Norwegian Regional Committee for Medical Research Ethics South-East-C approved the overall study and modifications (REK reference 2009/429). Signed, informed consent was obtained from participating women on behalf of themselves and their child. The NFFD trial was registered in ClinicalTrials.gov ID NCT01001689, date of registration: 27/10/2009. Children of mothers who participated in the NFFD and for whom dietary data was provided at 12 months of age, were included in the present analyses (*n* = 408).

The food frequency questionnaire was constructed in part using food frequency questions from the national young child dietary survey ‘Spedkost 2006–2007’ [18], with additional questions related to the purpose of the dietary intervention component of the NFFD study [19]. The questionnaire comprised 59 food and drink items, portion size of selected foods (8 items) and drink (4 items), and 13 items regarding meal patterns. For the present study, the following 11 components were chosen as indicators of diet quality: fruits (2 items), vegetables (3 items), fruit and vegetables combined (5 items), sweetened beverages (7 items), water (1 item), desserts and cake (5 items), total milk consumption (4 items), homemade meat- or fish- based dinners (2 items), commercially produced toddler’s dinner (3 items), homemade infant cereal (porridge) (1 item), and commercially produced infant cereal (1 item). All frequency questions related to foods and beverages had eight response options ranging from ‘never/less than weekly’ to ‘≥ five times/day’. The frequency of intake was recoded as follows: ‘never/less than every week’=0, ‘1–3 times/week’ = 0.3, ‘4–6 times/week’ = 0.7, ‘once/day’ = 1, ‘twice/day’ = 2, ‘three times/day’ = 3, ‘four times/day’ = 4, and ‘five times or more/day’=6). For food variables where multiple items contributed, we summed the recoded frequencies to yield total frequency of intake. The food frequency questions have shown strong test-retest reliability in a separate sample of one-year olds [19]. It has also been tested in a focus group discussion where mothers reported that the questionnaire enabled them, with few exceptions, to describe accurately what their child’s eating habits were [19].

Parents also responded to questions on how often the child had breakfast, lunch, dinner, supper, and between-meal snacks together with family, defined as eating the meal with at least one family member eating the same meal. The wording of the question was as follows: *How often does the child eat the following meals with another family member,* i.e.*, at the same time as at least one adult eats the same meal?* This definition of family meal is in line with the definition applied in the review by Verhage et al. [11], as *a social moment of the day during which food is eaten together with at least one family member*. Responses were reported as ‘never/less often than every week’, ‘1-3 times a week’, ‘4-6 times a week’ or ‘every day’. For description and analysis, frequency of eating with family was further dichotomized into seldom (ranging from ‘never’ to ‘three times a week’) or often (ranging from ‘four times a week’ to ‘every day’) for each family meal, respectively. We also computed a composite meal frequency score by summarizing each individual family meal scoring (seldom = 0, often = 1), yielding a possible score of frequent family meal participation ranging from 0 to 5.

The reliability of the family meal questions was assessed in a test-retest investigation carried out 1–2 weeks apart among 94 12-month-old children (45 male and 49 female children) [19]. Spearman’s r ranged from 0.59 (between-meal snacks with parent) to 0.73 (lunch with parent), while Cohen’s kappa ranged from 0.47 (between-meal snacks with parent) to 0.58 (breakfast with parent). The meal questions have not been validated against other data collection methods.

Mothers were asked about their highest level of education with the following response options; < 7 years of primary education; 7–10 years of primary education; trade school or 1–2 years of high school; completed high school; < 4 years at college/university, and ≥ 4 years at college/university. For analysis, educational level was dichotomized into low education (not having attended college or university) or high education (having attended college or university).

### Statistics

Statistical analyses were performed with SPSS for IBM statistical software package version 25.0 (IBM Corporation, Armonk, NY, USA). A two-sided *p*-value of < 0.05 was considered significant.

Maternal and child characteristics are presented with mean and standard deviation (SD) or proportions (%) as appropriate. Differences in diet between infants eating the respective main meals together with family often (4–7 times per week) as compared to seldom (3 times per week or less) were compared using independent sample t-test. We fit crude and adjusted linear regression models with frequency of eating with family (seldom vs. often) at breakfast, lunch, and dinner, respectively, as exposures, and frequency of intake of selected foods and beverages as outcome. We also estimated the dose-response association between increasing number of meals categories eaten with family often (0–5) and frequency of intake of the foods and beverages listed using linear regression models. Adjustments were made for randomization status, maternal level of education and child sex in all models.

Given that half of mothers in the study sample had been exposed to a lifestyle intervention during pregnancy we also present the relationship between family meal participation and child dietary intake according to maternal randomization status. In line with the aim of investigating potential differences in the relationship by maternal education attainment and child sex we also present all analyses stratified by maternal educational level (low vs. high education), and child sex (boy vs. girl), respectively. Regression coefficients with 95% confidence intervals (CI) are reported for all analyses. We assessed potential formal interaction of the relationship between family meal participation and child dietary intake by maternal education, randomization status, and child sex by including the following three interaction terms in the models (frequency of meals with family*education, frequency of meals with family*randomization, and frequency of meals with family*child sex).

Three of the diet variables were built from one dietary item with relatively few response options and a skewed distribution towards either low (home-made infant cereal) or high intake (water). We inspected the empirical association between aggregated meal category frequency and each diet variable to confirm reasonably linear associations, potential deviance from normal distribution and homoscedasticity of residuals. We inspected the empirical association between aggregated meal category frequency and each diet variable to confirm reasonably linear associations, potential deviance from normal distribution and homoscedasticity of residuals. In two instances, where the diet variables contained only one item with relatively few response options, skewed distribution towards either low (home-made infant cereal) or high intake (water) was observed, but because of relatively large sample size and low risk of biased estimates, linear regression was conducted.

## Results

Of the 591 women who took part in the original trial until birth of a singleton baby, 408 (69%) completed the food frequency questionnaire on behalf of their child at 12 months of age. A total of 51% belonged to the intervention group from the original trial, 75% of the mothers had higher education, 86% of mothers were currently employed, and 57% of the children were boys (Table 1). Approximately one in four children attended daycare at the time of completing the questionnaire.

**Table 1:** Demographics of women and children participating in the Norwegian Fit for Delivery study, and family meal participation characteristics provided 12 months post-partum (*n* = 408)

	*n*	%
**Maternal and child characteristics**		
Maternal education (university level)	305	74.8
Mother currently employed	352	86.3
Mother currently student	39	9.6
Maternal smoking (never)	302	70.4
Belonging to the NFFD intervention group	207	50.9
Child sex - proportion of boys	233	57.1
Child attending daycare	112	27.5
**Proportion of children eating the respective meals with family often**^**a**^		
Breakfast (*n* = 403)	297	73.7
Lunch (*n* = 398)	186	53.3
Dinner (*n* = 406)	302	74.4
Between-meal snacks (*n* = 379)	102	26.9
Supper (*n* = 401)	155	38.7
**Proportion of children having the respective number of meal categories with family often**^**a**^		
Never or seldom eating meals with family	36	9.6
One meal category	50	13.3
Two meal categories	82	21.9
Three meal categories	105	28.0
Four meal categories	51	13.6
Five meal categories	51	13.6

^a^ Family meals ‘often’ defined as 4–7 days per week

Based on parental report, a total of 74% of the 12-month-olds had breakfast with at least one family member often (Table 1). The proportion having family lunch and family dinner often were 53 and 74%, respectively, while 39% had supper and 27% between-meal snacks with family often.

In Table 2 crude and adjusted estimates of differences in dietary intake according to whether the respective meal categories were eaten with family seldom or often are presented. Children often participating in family breakfasts had less frequent intake of commercial infant cereal, and more frequent intake of water, in addition to slightly more frequent intake of desserts and cakes. Children often participating in family lunch had less frequent intake of commercial infant cereal and more frequent intake of home-made infant cereal and water. Children often participating in family dinner had more frequent intake of vegetables, home-made infant cereal, water, and milk, and less frequent intake of commercial dinners and commercial infant cereal. Children often participating in supper with family had less frequent intake of commercial infant cereal, as had children who often had between-meal snacks with family. Often having between-meal snacks with family was also associated with more frequent intake of vegetables and fruit and vegetable intake combined. Only minor differences between crude and adjusted estimates were observed.

**Table 2:** Differences in frequency of intake of food and drink items (times/day) according to eating the respective meals with family seldom vs. often^a^. Crude and adjusted^b^ regression coefficients (β) with 95% CI

	Breakfast with family often *n* = 297 (73.7%)				Lunch with family often *n* = 186 (53.3%)				Dinner with family often *n* = 302 (74.4%)				Between-meal snacks with family often *n* = 102 (26.9%)				Supper with family often *n* = 155 (38.7%)			
	Crude difference		Adjusted difference		Crude difference		Adjusted difference		Crude difference		Adjusted difference		Crude difference		Adjusted difference		Crude difference		Adjusted difference	
	β	95% CI	β	95% CI	β	95% CI	β	95% CI	β	95% CI	β	95% CI	β	95% CI	β	95% CI	β	95% CI	β	95% CI
Fruits	0.001	−0.288, 0.290	0.011	− 0.280, 0.301	0.067	− 0.190, 0.324	0.064	− 0.195, 0.322	− 0.125	− 0.414, 0.165	− 0.139	− 0.431, 0.152	0.117	−0.184, 0.417	0.111	−0.191, 0.413	−0.046	− 0.310, 0.217	−0.44	− 0.309, 0.221
Vegetables	0.249	− 0.032, 0.530	0.255	− 0.029, 0.538	0.206	− 0.045, 0.456	0.205	− 0.048, 0.457	**0.304**	**0.023, 0.585**	**0.300**	**0.016, 0.584**	**0.404**	**0.110, 0.697**	**0.408**	**0.112, 0.702**	0.214	−0.042, 0.471	0.226	−0.032, 0.484
Fruits and vegetables	0.250	−0.231, 0.732	0.265	−0.219, 0.750	0.273	−0.156, 0.702	0.268	−0.163, 0.700	0.179	−0.304, 0.662	0.160	−0.327, 0.648	**0.520**	**0.018, 1.023**	**0.519**	**0.014, 1.024**	0.168	−0.272, 0.608	0.182	−0.260, 0.624
Homemade dinners	−0.004	− 0.187, 0.179	− 0.011	− 0.195, 0.173	0.138	−0.025, 0.301	0.129	− 0.034, 0.293	0.177	−0.005, 0.360	0.179	−0.005, 0.363	0.117	− 0.076, 0.309	0.108	− 0.086, 0.301	0.092	−0.075, 0.258	0.096	−0.072, 0.263
Commercial dinners	−0.104	− 0.306, 0.99	− 0.094	−0.299, 0.110	− 0.093	− 0.274, 0.087	− 0.089	−0.270, 0.093	**− 0.235**	**− 0.437, − 0.033**	**−0.249**	**− 0.453, − 0.045**	−0.100	−0.308, 0.109	− 0.103	− 0.314, 0.107	− 0.041	− 0.226, 0.144	− 0.052	−0.238, 0.134
Homemade baby cereal	− 0.082	− 0.240, 0.077	−0.082	− 0.241, 0.077	**0.152**	**0.013, 0.292**	**0.154**	**0.014, 0.295**	**0.205**	**0.048, 0.363**	**0.197**	**0.036, 0.356**	−0.118	− 0.284, 0.047	− 0.123	−0.289, 0.043	− 0.095	−0.239, 0.048	− 0.088	− 0.232, 0.056
Commercial baby cereal	**−0.457**	**− 0.701, − 0.212**	**− 0.447**	**− 0.689, − 0.204**	**− 0.287**	**− 0.507, − 0.067**	**−0.286**	**− 0.504, − 0.069**	**−0.532**	**− 0.777, − 0.288**	**−0.550**	**− 0.792, − 0.308**	**− 0.334**	**−0.590, − 0.078**	**−0.313**	**− 0.566, − 0.059**	**−0.302**	**− 0.526, − 0.078**	**−0.276**	**− 0.498, − 0.053**
Drinking water	**0.516**	**0.182, 0.849**	**0.492**	**0.159, 0.825**	**0.523**	**0.229, 0.818**	**0.514**	**0.219, 0.808**	**0.590**	**0.257, 0.923**	**0.579**	**0.245, 0.912**	− 0.033	− 0.317, 0.382	0.043	− 0.307, 0.393	0.064	− 0.242, 0.370	0.094	− 0.212, 0.400
Milk	0.170	− 0.83, 0.423	0.169	−0.086, 0.424	0.019	−0.204, 0.242	0.029	−0.195, 0.253	**0.311**	**0.059, 0.564**	**0.304**	**0.050, 0.559**	0.166	−0.094, 0.427	0.148	−0.113, 0.409	0.191	−0.043, 0.425	0.181	−0.053, 0.416
Sweetened beverages	−0.207	− 0.460, 0.047	− 0.207	− 0.461, 0.047	− 0.121	− 0.357, 0.095	− 0.130	− 0.356, 0.097	− 0.192	−0.446, 0.062	− 0.198	−0.454, 0.057	−0.179	−0.441, 0.803	− 0.185	−0.449, 0.079	0.041	−0.271, 0.188	0.039	−0.271, 0.192
Desserts and cakes	**0.111**	**0.007, 0.216**	**0.111**	**0.007, 0.215**	0.056	−0.038, 0.150	0.059	−0.034, 0.153	0.101	−0.004, 0.206	0.105	0.000, 0.210	0.031	−0.075, 0.137	0.027	−0.080, 0.133	0.038	−0.057, 0.134	0.026	−0.069, 0.122

Significant results are bolded

^a^ Family meals ‘often’ defined as 4–7 days per week, and ‘seldom’ as 0–3 days per week

^b^ Associations adjusted for randomization status, maternal education, and child sex

Dose-response associations between overall family meal participation and dietary intake are presented in Table 3. For each additional meal category eaten with family often, frequency of vegetable intake increased by 0.15 times/day (*p* = 0.001), water by 0.17 times/day (*p* = 0.002), and milk by 0.09 times/day (*p* = 0.035). Commercial infant cereal was eaten less frequently with additional meals eaten with family, equivalent to 0.18 times less frequently/day (*p* < 0.001) (Table 3). There was no indication of confounding of these associations by maternal randomization, education level, or child sex.

**Table 3:** Dose-response association between number of meal categories (0–5) eaten with family often^a^ and dietary intake (times/day). Crude and adjusted^b^ regression coefficients (β) with 95% CI

	Crude model (*n* = 375)		Adjusted model (*n* = 372) ^b^	
	β	95% CI	β	95% CI
Fruits	0.013	−0.079, 0.104	0.011	−0.081, 0,103
Vegetables	**0.148**	**0.060, 0.237**	**0.148**	**0.059, 0.238**
Fruits and vegetables	**0.161**	**0.009, 0.313**	**0.159**	**0.006, 0.312**
Homemade dinners	0.058	0.000, 0.116	0.055	−0.003, 0.114
Commercial dinners	−0.059	− 0.122, 0.004	− 0.059	− 0.122, 0.005
Homemade baby cereal	0.010	− 0.040, 0.060	0.009	− 0.042, 0.059
Commercial baby cereal	**−0.182**	**− 0.258, − 0.106**	**−0.182**	**− 0.257, − 0.106**
Drinking water	**0.174**	**0.070, 0.279**	**0.170**	**0.065, 0.274**
Milk	**0.088**	**0.009, 0.166**	**0.085**	**0.006, 0.164**
Sweetened beverages	−0.073	−0.153, 0.007	−0.074	− 0.155, 0.006
Desserts and cakes	0.029	−0.003, 0.060	0.028	−0.004, 0.059

Significant results are bolded

^a^ Family meals ‘often’ defined as 4–7 days per week, and ‘seldom’ as 0–3 days per week

^b^ Associations adjusted for randomization status, maternal education, and child sex

Given that half of the mothers had participated in a lifestyle intervention while pregnant with their now 12-months-old, we checked for potential between-group differences in family meal participation and child dietary intake according to having been exposed to the intervention or not. There was no significant difference in frequency of family meals according to maternal randomization status (data not shown). Nor were there differences in child food intake according to maternal randomization (data not shown). Analyses stratified by randomization status are presented in Table 4. The inverse association between family meal frequency and commercial baby cereal was only evident in the intervention group (intervention group β − 0.284, 95% CI -0.389, 0.179, *p* < 0.001 vs. control group β − 0.079, 95% CI -0.187, 0.029, *p* = 0.153) (interaction term *p* = 0.007), as was the association between increasing number of family meals and frequency of intake of water (intervention group β 0.274, 95% CI 0.118, 0.430, *p* = 0.001 vs. control group β − 0.060, 95% CI -0.079, 0.198, *p* = 0.394) (interaction term 0.063). The effect size of the associations with milk and homemade dinners were also larger, and significant only in the intervention group.

**Table 4:** Dose-response association between number of meal categories (0–5) eaten with family often^a^ and dietary intake (times/day). Crude and adjusted^b^ regression coefficients (β) with 95% CI. Analyses stratified by randomization status

	Adjusted model confined to the control group (*n* = 182)		Adjusted model confined to the intervention group (*n* = 190)	
	β	95% CI	β	95% CI
Fruits	0.004	− 0.125, 0.133	0.017	− 0.117, 0.150
Vegetables	**0.135**	**0.001, 0.268**	**0.169**	**0.048, 0.289**
Fruits and vegetables	0.139	−0.095, 0.372	0.185	−0.018, 0.389
Homemade dinners	0.040	−0.063, 0.142	**0.077**	**0.018, 0.136**
Commercial dinners	−0.016	− 0.076, 0.043	− 0.100	− 0.213, 0.012
Homemade baby cereal	− 0.010	− 0.094, 0.074	0.028	− 0.031, 0.086
Commercial baby cereal	− 0.079	− 0.187, 0.029	**− 0.284**	**− 0.389, − 0.179**
Drinking water	0.060	− 0.079, 0.198	**0.274**	**0.118, 0.430**
Milk	0.074	−0.053, 0.202	**0.101**	**0.006, 0.196**
Sweetened beverages	−0.059	−0.190. 0.072	− 0.087	−0.185, 0.011
Desserts and cakes	0.025	−0.008, 0.058	0.034	−0.021, 0.089

^a^ Family meals ‘often’ defined as 4–7 days per week, and ‘seldom’ as 0–3 days per week

^b^ Associations adjusted for maternal education and child sex. Significant results are bolded

Associations between number of meal categories eaten with family often and dietary intake stratified according to maternal education are presented in Table 5. There was no difference in frequency of having family meals according to maternal education (data not shown). Children of mothers with higher maternal education had more frequent intake of commercial infant cereal and less frequent intake of dessert and cake compared to children of mothers with lower level of education (data not shown). An inverse association between number of meal categories eaten with family often and frequency of intake of sweetened beverages was only observed among children of mothers with lower education (lower education β − 0.200, 95% CI -0.334, − 0.064, *p* = 0.004 vs. higher education β − 0.028, 95% CI -0.127, 0.071, *p* = 0.584) (interaction term *p* = 0.001) (Table 5). The association with vegetables (*p* = 0.010) and water intake (p = 0.004) was only significant among children of mothers with higher education.

**Table 5:** Dose-response association between number of meal categories (0–5) eaten with family often^a^ and dietary intake (times/day). Crude and adjusted^b^ regression coefficients (β) with 95% CI. Analyses stratified by maternal education^c^

	Adjusted model confined to lower maternal education^c^ (*n* = 91)		Adjusted model confined to higher maternal education^c^ (*n* = 281)	
	β	95% CI	β	95% CI
Fruits	0.074	−0.108, 0.255	− 0.018	− 0.127, 0.090
Vegetables	0.137	−0.034, 0.309	**0.141**	**0.034, 0.247**
Fruits and vegetables	0.211	−0.088, 0.510	0.123	−0.060, 0.305
Homemade dinners	0.075	−0.004, 0.154	0.045	−0.030, 0.120
Commercial dinners	−0.094	− 0.227, 0.039	− 0.049	−0.123, 0.025
Homemade baby cereal	− 0.037	− 0.025, 0.099	− 0.002	− 0.067, 0.064
Commercial baby cereal	**−0.194**	**− 0.338, − 0.049**	**−0.178**	**− 0.268, − 0.088**
Drinking water	0.158	− 0.061, 0.376	**0.177**	**0.056, 0.298**
Milk	0.130	−0.050, 0.311	0.064	−0.024, 0.152
Sweetened beverages	**−0.200**	**−0.334, − 0.064**	−0.028	− 0.127, 0.071
Desserts and cakes	0.054	−0.046, 0.154	0.023	−0.003, 0.049

^a^ Family meals ‘often’ defined as 4–7 days per week, and ‘seldom’ as 0–3 days per week

^b^ Associations adjusted for randomization status and child sex

^c^ Higher education defined as university education of ≥4 years and lower education as < 4 years of university education

Finally, there was no difference in frequency of family meal participation according to child sex (data not shown). Girls had more frequent intake of homemade dinner, but no difference was observed for the other investigated foods and beverages (data not shown). Most associations between number of meal categories eaten with family often and food intake were only significant among in boys, i.e., vegetable intake (*p* = 0.002), fruits and vegetables intake combined (*p* = 0.047), home-made dinners (*p* = 0.029), and intake of water (p = 0.002) (Table 6).

**Table 6:** Dose-response association between number of meal categories (0–5) eaten with family often^a^ and dietary intake (times/day). Crude and adjusted^b^ regression coefficients (β) with 95% CI. Analyses stratified by child sex

	Adjusted model confined to boys (*n* = 215)		Adjusted model confined to girls (*n* = 157)	
	β	95% CI	β	95% CI
Fruits	0.011	−0.105, 0.128	0.005	−0.148, 0.158
Vegetables	**0.172**	**0.056, 0.278**	0.112	−0.042, 0.267
Fruits and vegetables	**0.183**	**0.002, 0.364**	0.117	−0.152, 0.386
Homemade dinners	**0.054**	**0.005, 0.103**	0.060	−0.061, 0.181
Commercial dinners	−0.074	−0.173, 0.024	− 0.051	−0.127, 0.025
Homemade baby cereal	0.012	−0.042, 0.065	0.004	−0.091, 0.100
Commercial baby cereal	**−0.198**	**−0.296, − 0.099**	**−0.161**	**− 0.282, − 0.040**
Drinking water	**0.231**	**0.083, 0.380**	0.110	−0.039, 0.259
Milk	0.058	−0.088, 0.205	0.098	−0.70, 0.266
Sweetened beverages	−0.033	−0.123, 0.057	− 0.123	−0.271, 0.025
Desserts and cakes	0.037	−0.014, 0.087	0.026	−0.008, 0.061

Significant results are bolded

^a^ Family meals ‘often’ defined as 4–7 days per week, and ‘seldom’ as 0–3 days per week

^b^ Associations adjusted for randomization status and maternal education

## Discussion

In the present study we observed dietary and potential nutritional benefits of frequent participation in family meals as early as 12 months of age, specifically more frequent intake of vegetables, homemade infant cereal, milk, and water, and less frequent intake of commercial infant foods. Combined, these findings indicate improved diet quality with regular participation in family meals, with dinner participation being associated with more dietary benefits than the other meal categories. We also observed a dose-response association between number of family meal categories eaten often and child dietary intake. Some of the associations differed according to maternal randomization status in a previous lifestyle intervention. Other associations differed according to maternal education or child sex. To our knowledge, this is the first study to investigate potential differences in the relationship of early family meal participation with child dietary intake according to maternal education and child sex.

Regarding specific meal categories, dinner and between-meal snacks with family were more strongly associated with vegetable intake than were the other meals. This is not surprising, given that vegetables are more typically served with dinner than with the other meals in Norway. Between-meal snacks may also be an opportunity for vegetable intake. The dose-response association between family meal participation and child vegetable intake did not differ according to maternal randomization status. The effect size of the association was similar across maternal education, but not significant in children of low educated mothers. For girls, the association was weaker than for boys, and not significant. Frequency of fruit intake was, however, neither associated with specific meals, nor overall family meal frequency. This contrasts with the findings of Fitzpatrick et al. [12] who found that the odds of serving both fruit and vegetables increased by 15 and 14%, respectively, for each night the family ate dinner together. The lack of association with fruit intake in the present study could be due to culture-specific meal patterns and indicate that offering fruit to infants and toddlers may be less dependent upon meal structure than many other foods in the Norwegian context.

More frequent homemade vs. commercially produced dinners, and more frequent intake of vegetables with participation in more family meal categories, could imply that one-year-olds eating with family are approaching family diet earlier, possibly with less need for commercial infant foods. It should be noted that commercial infant foods are not necessarily less nutritious than home-made foods, especially not commercial infant cereal that are normally fortified with iron and other nutrients. Prolonged use of commercial infant and toddler foods may, however, fail to support normal eating development, potentially delaying acceptance of age-appropriate, and more nutrient-dense family foods due to their homogenous taste and texture [20]. Less frequent intake of commercial infant cereal combined with slightly more frequent intake of homemade infant cereal with regular participation in more family meal categories, support this notion.

Some of the observed associations differed according to whether the mother had participated in the previously described lifestyle intervention during pregnancy or not. Associations between participating in more family meal categories and the various aspects of child dietary intake tended to be stronger in children of mothers in the intervention group, in which more family meal participation was significantly associated with more frequent intake of vegetables, milk, water, and homemade dinners, and with less frequent intake of commercial baby cereal (Table 4). This indicates an indirect effect of the previous maternal intervention on feeding practices or family diet even though there was no intervention-related between-group difference regarding child diet or family meal participation at child age 12 months. The lifestyle intervention that mothers in the intervention group attended during pregnancy, focused, among others, on structured meals, including vegetables with dinner, and preparing more meals from whole foods rather than using ultra-processed foods [21]. The intervention proved effective in improving maternal dietary behaviors during pregnancy, among them increased fruit and vegetable intake and intake of water [22]. It is conceivable that there could be sustained effect for some of these dietary behaviors post-partum, which could benefit the child when included in family meals. There is reason to believe that the control group represent the general population to a larger degree than the intervention group in this study and that more frequent intake of vegetables is the most robust association with frequent participation in family meals.

Family meal frequency was similarly associated with child dietary intake regardless of maternal education, except for an inverse association with sweetened beverages that was confined to girls of mothers with lower education. It should be noted that the number of children with low educated mothers were small, yielding substantially less statistical power to detect significant associations. Effect sizes were larger for several of the associations in the low education group, leaving room for speculation about a potential larger nutritional benefit of family meal participation in this group.

The positive association between frequency of family meals and vegetable intake, home-made dinners, and drinking water was confined to boys and might indicate that the family meal setting in this sample was more conducive to intake of these food items for boys than for girls (Table 6). Bouhlal et al. [23] found differences in maternal dietary choices made for boys and girls, favoring more calories for boys and healthier food choices for girls than for boys while no gender difference in feeding practices according to child gender were found by Hendy & Williams [24]. None of these studies are directly comparable to our findings due to older age of the children and no relationship to the family meal setting. Given mounting evidence of sex-differences in early-life health and nutritional vulnerability, potential sex-specific differences in meal-related parental feeding practices, family meal characteristics and nutritional consequences, gender aspects should be further explored in future family meal research [15].

Overall, the family meal context holds potential for improved diet quality, regardless of maternal previous experience, educational attainment, and child sex. Several aspects make our findings relevant to 12-months-olds’ nutritional short- and long-term health. One of the strongest associations of regularly eating with family was the frequency of intake of vegetables. This aligns with previous research among older children [8–10]. Vegetables provide vitamins, antioxidants, minerals, and soluble dietary fiber at a low energy yield. Combined with more frequent intake of water and milk, the nutrient density of child diet seemed to increase with increasing family meal participation in the present study. The family meal setting may facilitate child familiarization of new foods, tastes, and textures. Observational learning and role-modeling by family members may thus promote food acceptance. Eating with family as compared to being fed outside family meals may also imply more varied food exposure and promote self-feeding skills because caregivers in the family meal setting are likely to be occupied with their own eating. The opportunity to self-feed may promote child autonomy and self-regulation of food intake that is highly important to long-term nutritional health [3].

Parents play a major role in setting rules and expectations, making decisions on the timing and content of child diet, and providing role modelling [25]. Encouragement and positive feedback during meals could help prevent problems related to food and eating and contribute to establishing healthy eating habits from early childhood [26].

Orchestrating family meals requires planning and preparation of food. Swanson et al. [13] found that family meals was highly correlated with frequency of cooking meals from scratch in a sample of socially disadvantaged mothers. Our findings support an association between family meal participation and less frequent use of commercial infant foods. Child eating development and willingness to try new food may be hampered by delayed introduction to lumpy foods as may happen with strong reliance on pureed commercial baby foods [27]. Emmett et al. [27] found that 15 months old children eating with their mother and eating the same as their mother were less likely to exhibit picky eating, while feeding ready-prepared food was predictive of picky eating.

The Norwegian Directorate of Health’s report “Food and meals for infants” claims that sharing meals has the potential to create happiness, belonging and wellbeing, and further, that a positive atmosphere and adequate time for eating lead to positive experiences, stimulate appetite and promote learning and socialization [26]. Even though parental ability to create a positive meal atmosphere is likely to vary, we believe that our findings underpin the health potential of including the child in family meals as early as possible. Walton et al. [28] describe how parents’ perceptions of family meals are influenced by their past experiences in childhood and early adulthood and show that a ‘family togetherness’ orientation to meals experienced in childhood is predictive of applying the same orientation during parenthood. Based on their findings, early family meal participation may even be influential in a generational perspective.

### Strengths and limitations

To our knowledge, this is the first study to investigate the relationship between family meal participation and dietary intake confined to 12-month-olds. The separate investigation of dietary associations with each family meal category, and the dose-response investigation of all meal categories combined also address gaps in the family meal literature. Other strengths to the present study are the high participation rate in the original NFFD trial from which data was used, and detailed dietary data from a large proportion of the children born to mothers in the study. As only nulliparous women were included in the trial, all children in our dataset are first-borns to the mother and are therefore likely to be sharing meals mainly with adults. Data was provided for all children at or around 12 months of age This makes the sample of children more homogenous and precludes potential confounding and effect modification due to role-modeling by older siblings or child age. The separate reporting for five family meal categories made it possible to investigate associations for breakfast, lunch, dinner, supper, and between-meal snacks as separate exposures besides investigating dose-response associations of additional meal categories eaten with family often.

There are also limitations that warrant discussion. The associations presented are cross-sectional in nature and causality cannot be inferred. For the investigation of family meal regularity, we dichotomized family meal frequency as seldom (0–3 times per week) or often (4–7 times per week) for each meal category, respectively. This was a forced choice, given that family meal frequency was limited to four response options in the questionnaire [19]. We do believe that this categorization for a given meal category is meaningful and facilitates valid comparisons of the relationship between family meals and dietary intake. From this crude categorization it follows, however, that a child who had every meal category 3 days per week would be categorized with the lowest score, while a child who had every meal category 4 days per week would be classified with the highest family meal score. To ascertain more precision and larger variance in the family meal participation measure, analyses using a weekly family meal frequency score as was done by Litterbach et al. [29], might be an option in future analyses.

The number of daily meals vary between families. Having fewer daily meals overall, precluded attainment of the highest meal score in this study even if every daily meal was eaten with family. Hence, the highest family meal scoring may not necessarily reflect a more beneficial feeding practice than a slightly lower score. Given that the outcome of interest in relation to family meal frequency was frequency of intake of selected food groups, the chosen operationalization may indirectly describe associations with more frequent daily meals. Most 12-month-olds eat more than three meals a day, and we therefore believe that the analyses are valid. As variation in cut-offs and definitions of family meals complicate comparison across studies, the family meal literature would benefit from a standardization of terms and definitions [9–11].

Fair (Cohen’s kappa) to high (Spearman’s correlation) test-retest reliability has been documented for the family meal questions [19], but the meal questions have not been validated against other methods of data collection. Some degree of misclassification of true family meal participation are therefore likely to have occurred. Given a true association between family meal participation and dietary intake, reporting higher than actual family meal participation would tend to underestimate associations with dietary intake. The same holds true with reporting lower than actual family meal participation, as this would tend to overestimate associations in the lower family meal participation range. Misclassification would therefore tend to bias the observed associations towards the null. From this we believe that the reported estimates are conservative. We have no information regarding meal practices for the lunch meal for the quarter of children who attended daycare. Children in daycare may have the same benefits of daycare meal participation as if the meals were eaten with family. If this be the case, failing to account for daycare meals in the family meal score may also have biased our estimates. Further investigation into daycare mealtime practices and whether these act as proxy for family mealtimes and therefore should be included in such classifications, is warranted.

Assumptions for linear regression were reasonably met for all dietary variables except for water. Most children had a high intake of water, and the estimated difference according to family meal participation may not be nutritionally relevant. Given that the estimates for dietary intake across family meal frequency were based on relatively crude categorization of frequency of intake, the observed associations should be taken as indications rather than exact measures.

We cannot exclude residual or unmeasured confounding of the presented associations. Parents regularly including their child in family meals may have stronger awareness of the importance of both shared meals and child nutritional needs. They may also be more interested in diet, have healthier diet themselves, and give higher priority to the preparation of meals in everyday life. Higher parental self-efficacy for meal preparation may translate into more cooking from raw ingredients and more balanced meals [30]. The frequency of including the child in the family meals could be a surrogate measure of consciousness regarding health, diet, and dietary behavior in general, which could imply that variation in parental traits not necessarily captured by the sociodemographic variables could confound associations. We have, however, provided an indication that parents’ ability to orchestrate family meals seem to provide an opportunity for healthier foods to be eaten by the 12-month-olds.

Mothers in the present study were predominantly white, European, highly educated, and first-time mothers, and therefore not fully representative of the background population. This also applies to their children. Even so, we found dietary differences according to how often the children were fed in a family meal context. A larger and more representative sample in future studies might provide larger variability in family meal participation and larger dietary variability. To establish causal associations between early family meal practices and long-term health, more knowledge from intervention studies with longitudinal follow-up is needed.

## Conclusion

Our findings support beneficial associations between participation in family meals and child dietary intake at 12 months of age. The observed differences in dietary intake between children eating most daily meals in a family setting and those mainly fed outside family meals could be important in a public health perspective. Our findings extend previous research by showing that family meal participation in general is associated with healthy eating in children as young as 12 months of age and by showing that the relationship with some of foods and beverages differed according to maternal education and child sex. Encouraging eating together as a family from early childhood and empowering parents in planning and preparing healthy family meals with appropriate adjustments for their infant or toddler, may structure the food and eating environment, improve child dietary intake, and enhance nutritional health for both children and parents.

## Data Availability

The datasets analysed during the current study are available from the corresponding author on reasonable request.
